# Aberrant Epithelial Cell Proliferation in Peripheral Airways in Bronchiectasis

**DOI:** 10.3389/fcell.2020.00088

**Published:** 2020-02-20

**Authors:** Yang Peng, Ai-ru Xu, Shi-ying Chen, Yan Huang, Xiao-rong Han, Wei-jie Guan, De-Yun Wang, Nan-shan Zhong

**Affiliations:** ^1^State Key Laboratory of Respiratory Disease, National Clinical Research Center for Respiratory Disease, Guangzhou Institute of Respiratory Health, First Affiliated Hospital of Guangzhou Medical University, Guangzhou Medical University, Guangzhou, China; ^2^Department of Otolaryngology, Yong Loo Lin School of Medicine, National University of Singapore, Singapore, Singapore

**Keywords:** bronchiectasis, progenitor cells, epithelium, bronchiole, hyperplasia

## Abstract

Dilation of bronchi and bronchioles caused by destruction and excessive epithelial remodeling is a characteristic feature of bronchiectasis. It is not known how epithelial progenitor cells contribute to these pathologic conditions in peripheral airways (bronchioles) in bronchiectasis. We aimed to explore the expression levels of signature airway progenitor cells in the dilated bronchioles in patients with bronchiectasis. We obtained the surgically resected peripheral lung tissues from 43 patients with bronchiectasis and 33 control subjects. Immunostaining was performed to determine the expression patterns of thyroid transcription factor-1 (TTF-1, for labeling progenitor cells in distal airways), P63 (basal cells), club cell 10 kDa protein (CC10, club cells), and surfactant protein C (SPC, alveolar type II epithelial cells) in epithelium or sub-epithelium. Here, we reported significantly lower percentage of TTF-1^+^ cells and CC10^+^ cells, and higher percentage of P63^+^ cells within the epithelium of dilated bronchioles compared with control bronchioles. In airway sub-epithelium of the dilated bronchioles, epithelial hyperplasia with disarrangement of TTF-1^+^ cells yielded cuboidal (100%) and columnar (93.0%) type among bronchiectasis patients. Most progenitor cell markers co-localized with TTF-1. The median (the 1st, 3rd quartile) percentage of P63^+^TTF-1^+^, CC10^+^TTF-1^+^, and SPC^+^TTF-1^+^ cells was 16.0% (8.9, 24.0%), 14.5% (7.1, 20.8%), and 52% (40.3, 64.4%), respectively. For cuboidal epithelial hyperplasia, 91.0% (86.5, 94.0%) of areas co-stained with SPC and TTF-1. Columnar epithelial hyperplasia was characterized by TTF-1 co-staining with P63^+^TTF-1^+^ and CC10^+^TTF-1^+^ cells. Taken together, aberrant proliferation of airway progenitor cells in both epithelium and sub-epithelium are implicated in bronchiectasis.

**Graphical Abstract:**
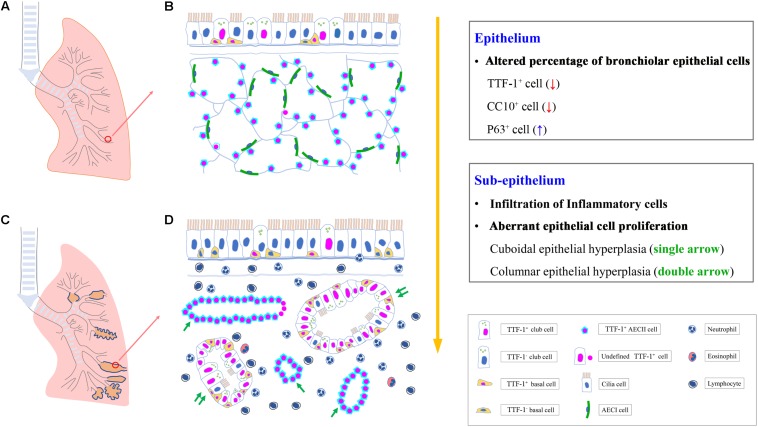
Aberrant proliferation of airway progenitor cells in both epithelium **(A,B)** and sub-epithelium **(C,D)** in peripheral airways in bronchiectasis. AECI, type I alveolar epithelial cell; AECII, type II alveolar epithelial cell; CC10, club cell 10 kDa protein; TTF-1, thyroid transcription factor-1.

## Introduction

Bronchiectasis is characterized by recurrent infections and heightened inflammatory responses that are responsible for progressive lung injury and irreversible dilatation of bronchi and bronchioles (Boyton and Altmann, 2016). In many cases, the pathologic dilatation of bronchioles may co-exist with bronchiectasis of the large to medium-sized airways. Despite the vicious cycle model, the pathophysiology of bronchiectasis remains poorly understood. Impaired mucociliary clearance and aberrant repair (including hyperplasia) of airway epithelium has recently been implicated in bronchiectasis (Chen et al., 2018). However, few effective therapies are available to bronchiectasis management.

Advances in pathophysiology have provided fundamental insights into the role of airway epithelium in the milieu of recurrent infections and inflammation. Endogenous airway progenitor cells are crucial to lung homeostasis and regeneration because of their self-renewal capacity through differentiation into normal airway epithelial cells (ECs) (Nikolić et al., 2018; Liu et al., 2019). Abnormalities of progenitor cells have been associated with airway inflammatory diseases. For instance, basal cells (BCs) counts were lower and self-renewal capacity was impaired in chronic obstructive pulmonary disease (COPD) (Ghosh et al., 2018). Identification of the critical drivers (particularly the identity and sub-populations of airway progenitor cells) would help unravel the mechanisms of pathogenesis from a novel perspective and explore targets for therapeutic interventions for bronchiectasis (Nikolić et al., 2018).

The primordial lung bud was first demarcated with the expression of thyroid transcription factor-1 (TTF-1, also known as Nkx2 homeobox 1) (Whitsett et al., 2015). TTF-1 is a “master gene” in maintaining lung morphogenesis and cytodifferentiation of certain EC lineages (Akram et al., 2016). TTF-1 has been regarded as the earliest known marker of the lung epithelial cell lineage, and is expressed in all epithelial lineages of the lower respiratory tract during development (Kotton and Morrisey, 2014). It has also been reported that the respiratory lineage initiates from the differentiation of TTF-1-positive progenitor cells that ultimately form the gas-exchange surface (Sui et al., 2019). In human lungs, TTF-1 is required for expression of several epithelial markers in the distal developing airways, including BCs and club cells of the distal airways, and alveolar type II EC (AECII) (Hösgör et al., 2002; Hawkins et al., 2017; Wang G. et al., 2019). When subject to injury, these ECs must be replaced rapidly to maintain normal lung structure and function. BCs play a key role in the maintenance of normal airway epithelial architecture through self-renewal (differentiation into ciliated and club cells), whereas club cells (formerly known as Clara cells) and AECII are largely responsible for bronchiolar and alveolar repair, respectively (Walters et al., 2013; Hannan et al., 2015; Liu et al., 2019). However, there has not been a systematic investigation directly linking the abnormality of airway progenitor cells to bronchiectasis.

We hypothesized that abnormality of airway progenitor cells that led to failure of airway injury repair would be key to bronchiectasis pathogenesis. Here, we sought to investigate the spatial distribution and quantify airway progenitor cells with histologic assessment of specific markers, including TTF-1^+^ cells for multiple airway progenitors, P63^+^ for BCs, Club Cell 10 kDa Protein (CC10)^+^ for club cells and surfactant protein C (SPC)^+^ for AECII in the bronchiolar or alveolar epithelium of distal airways in bronchiectasis (Rawlins et al., 2009; Mou et al., 2012; Crystal, 2014; Jacob et al., 2017). We have identified two major patterns of epithelial hyperplasia (cuboidal or columnar) which can be extensively labeled by TTF-1^+^ in sub-epithelium of the dilated bronchioles. Our results provide a basis for promoting EC repair through “normalizing” airway progenitor cells, which might help reverse the trend of bronchiectasis progression.

## Materials and Methods

### Patient Recruitment

**FIGURE 1:**
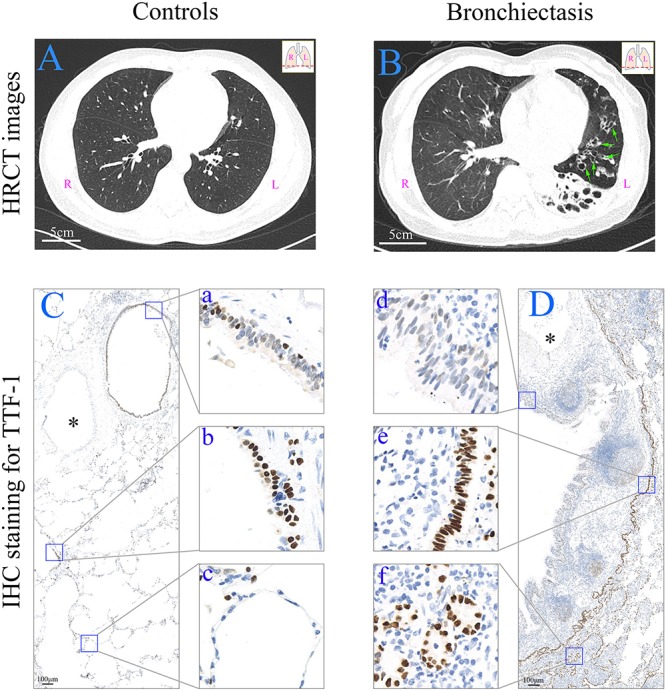
HRCT scan images and expression of TTF-1 protein determined by means of immunohistochemistry in the peripheral lung tissue. **(A)** HRCT scan image from a control subject (age: 45; sex: male). Scale bar = 5 cm. **(B)** HRCT scan image from a patient with bronchiectasis [age: 65; sex: female; HRCT score: 8; dilated bronchioles in the peripheral left lower lobe (green arrows)]. Scale bar = 5 cm. **(C,D)** In a control subject (from a donor) **(C)**, TTF-1^+^ cells are present in bronchiole (a), respiratory bronchiole (b) and alveoli (c), respectively. In a patient with bronchiectasis (age: 14; sex: male; HRCT score: 3) **(D)**, TTF-1^+^ cells are decreased in the epithelium of the dilated bronchioles (d), and both columnar (e) and cuboidal (f) epithelial hyperplasia of TTF-1^+^ cells are present. The similar accompanying vessel are shown in ^∗^. Scale bar = 100 μm. HRCT: high-resolution computed tomography; IHC: immunohistochemistry; L: left side; R: right side; TTF-1: thyroid transcription factor-1.

Study protocol approval was obtained from the institutional review boards of The First Affiliated Hospital of Guangzhou Medical University. All study participants provided informed consent. The diagnosis of control subjects with benign tumor and patients with bronchiectasis was based on chest high-resolution computed tomography (HRCT) (Figures 1A,B) and the documentation of respiratory symptoms (including chronic cough and sputum production) from medical charts. The modified Reiff score was recorded to evaluate the radiologic severity of bronchial dilatation (tubular: 1 point, varicose: 2 points, cystic: 3 points), with the maximal score of 18 for six lobes (with the lingula lobe being a separate lobe) (Chalmers et al., 2014). The site of bronchiectasis was categorized as central, peripheral, or mixed (central + peripheral) airways.

The duration of respiratory symptoms (particularly cough) and the most recent spirometry findings [performed according to international standards (Miller et al., 2005)] were extracted from medical charts. Cystic fibrosis was not routinely screened because of the extremely low prevalence in China. Smokers were defined as current cigarette smokers if they had smoked for more than 10 pack years. No study participant had a physician’s primary diagnosis of asthma.

We evaluated peripheral lung tissues with the dilated bronchioles from 43 bronchiectasis patients and peripheral normal lung tissues from 33 controls subjects [including 20 peripheral lung tissues from healthy donors, 13 adjacent normal tissue (>1 cm away from the affected tissue) from subjects with benign tumor (six with pulmonary hamartoma, five with atypical adenomatous hyperplasia and two with intrapulmonary lymph nodes)] who underwent lung transplantation, lobectomy or pneumonectomy between June 2017 and June 2019. Because of the policy on anonymization of data from healthy donors, the clinical characteristics of control subjects could only be retrieved from 13 patients with benign tumor (Table 1).

**TABLE 1:** Baseline characteristics of study participants.

	**Control**	**Patients with**
	**subjects**	**bronchiectasis**
Subjects, *n*	33	43
Healthy donors, *n* (%)	20 (60.6)	–
Patients with benign tumor, *n* (%)^†^	13 (39.4)	–
Sex (M/F), *n*	6/7	14/29
Age, years	48.9 ± 13.5	47.4 ± 14.6
Smokers, *n* (%)	0 (0)	3 (7.0)
BMI (kg/m^2^)^‡^	22.3 ± 2.7	21.3 ± 2.8
FEV_1_ % predicted^‡^	105.6 ± 12.9	81.8 ± 23.9
FEV_1_/FVC (%)^‡^	83.4 ± 5.1	78.3 ± 10.6
Duration of disease, years	–	6.3 ± 8.0
Modified Reiff score of HRCTs	–	6.3 ± 4.3
**The percentage of inflammatory cells^§^**		
Eosinophils	–	8.3 ± 5.9
Neutrophils	–	9.8 ± 5.0
CD4^+^ T cell	–	33.1 ± 9.7
CD8^+^ T cell	–	15.8 ± 7.5
**Inflammatory cell infiltration, *n* (%)^¶^**		
Eosinophilic infiltration		20 (46.5)
Neutrophilic infiltration		19 (44.2)

*^†^The characteristics of control subjects were recorded from patients with benign tumor (*n* = 13). ^‡^These information from four patients with bronchiolectasis were miss. ^§^ Differential cell counts of the inflammatory cells were expressed as the percentage number of positive staining cells/200 leukocytes × 100%. ^¶^ Eosinophilic or neutrophilic infiltration is categorized by each inflammatory cell count being greater than 10% of the total leukocyte count. The data of age, BMI, FEV_1_% predicted, FEV_1_/FVC (%), duration of disease, modified Reiff score of HRCTs and the percentage of inflammatory cell were presented as mean ± standard deviation. BMI, body mass index; F, female; FEV_1_ = forced expiratory volume in 1 s; FVC, forced vital capacity; HRCT, high-resolution computed tomography; M, male.*

### Tissue Preparation, Immunohistochemistry, and Immunofluorescent Staining

Three-micrometer thick tissue sections were dewaxed in xylene, rehydrated in graded alcohols, and rinsed in distilled water. Sections were then subject to heat-induced antigen retrieval in Tris–EDTA buffer (pH 9.0) at 95°C for 15 min and cooled at room temperature.

For immunohistochemistry (IHC), staining of TTF-1 (1:300, anti-rabbit, ab76013, Abcam, United States), CD4 (1:200, anti-rabbit, ab133616, Abcam, United States), CD8 (1:50, anti-mouse, ab17147, Abcam, United States) and neutrophil elastase (1:600, anti-mouse, clone NP57, Dako A/S, Glostrup, Denmark) was performed with a modified horseradish peroxidase (HRP) technique with Dako Cytomation EnVision 1 System-HRP (Dako A/S). Endogenous peroxidase activity was blocked with 0.3% hydrogen peroxide. Sections were stained with primary antibody at 4°C for overnight incubation. Slides were incubated with Dako EnVision 1 System-HRP (Dako A/S) at room temperature for 30 min, followed by the addition of HRP substrate (diaminobenzidine), and counterstained with hematoxylin. Images were obtained with digital pathological section scanner (PRECICE500B, Beijing, China).

For immunofluorescent (IF) staining, sections were incubated with primary polyclonal antibodies of P63 (1:50, anti-mouse, ab735, Abcam, United States), CC10 (1:500, anti-mouse, sc-365992, Santa Cruz Biotechnology, United States) and SPC (1:100, anti-mouse, sc-518029, Santa Cruz Biotechnology, United States), respectively. We performed triple co-staining with TTF-1 (1:300, anti-rabbit, ab76013, Abcam, United States), α-tubulin (1:100, anti-chicken, ab89984, Abcam, United States) and one of the above-mentioned markers for overnight incubation at 4°C, followed by incubation with Alexa Fluor 488-, Alexa Fluor 555-, and Alexa Fluor 647-conjugated secondary antibodies (1:500, Life Technologies, Carlsbad, CA, United States) at 37°C for 1 h. The nuclei were visualized by staining with 4′-6-diamidino-2-phenylindole (Life Technologies, Carlsbad, CA, United States). Images were acquired with fluorescence microscopy (Leica DM6, Wetzlar, Germany).

For negative controls, primary antibodies were substituted with species- and subtype-matched antibodies of the same concentration.

### Morphometric Analysis

In control subjects, the peripheral airway bronchioles were defined as having the diameter of <2 mm (small airways), and consist of: (1) proximal bronchioles: 1–2 mm in diameter; (2) distal bronchioles: <1 mm in diameter; (3) terminal bronchioles: small non-alveolarized bronchioles in the vicinity of respiratory bronchioles; (4) respiratory bronchioles: alveolarized bronchioles (Okuda et al., 2019). Because of the difficulty to differentiate proximal and distal from terminal bronchioles solely based on the airway diameters (readily subject to artifacts associated with post-fixation processing), we specifically focused on the inclusion criteria for normal bronchioles as follows: (1) non-alveolarized bronchioles 0.3–2 mm in diameter; (2) a narrow collapsible lumen, lined with simple columnar ciliated epithelium without cartilage plates and sub-epithelial glands.

In patients with bronchiectasis, the dilated non-alveolarized bronchioles were defined as: (1) the non-alveolarized bronchioles >2 mm in diameter, and presented with inflammatory cell infiltration surrounding the sub-epithelium; (2) with pseudostratified ciliated columnar or simple columnar ciliated epithelium without cartilage plates and sub-epithelial glands; (3) with a diameter larger than the accompanying thick-walled vessel.

### Marker Expression Profiling of Bronchiolar Epithelium

The total number of ECs was assessed by manually counting all cellular nuclei located at epithelial areas. We specifically enumerated all ECs, TTF-1^+^ cells, P63^+^ BCs and CC10^+^ club cells. The percentage of TTF-1^+^, P63^+^ and CC10^+^ cells was calculated as the number of positively stained cells divided by 200 ECs multiplied by 100%. Five to ten areas of epithelium staining from tissue sections were taken randomly from each sample in a blinded manner at high power fields (HPFs).

### Marker Expression Profiling of Sub-Epithelium

The total number of abnormal hyperplastic progenitor cells in sub-epithelium was assessed by counting all TTF-1^+^ cells with cuboidal or columnar epithelial hyperplasia. Ten areas with abnormal TTF-1^+^ epithelial hyperplasia (either cuboidal or columnar) were randomly counted for each sample (*n* = 43) in 5∼10 HPFs in a blinded manner (Supplementary Figure S2). We calculated the percentage of cuboidal and columnar epithelial hyperplasia (total *N* = 430). Most areas of sub-epithelial hyperplasia in the dilated bronchioles can be extensively labeled with TTF-1^+^, most of which co-stained with P63, CC10 or SPC. The fluorescence intensity was not analyzed because it was highly influenced by the quality of the material. Therefore, the percentage of P63^+^, CC10^+^ and SPC^+^ cells were expressed as the percentage of positively stained cells divided by 200 sub-epithelial TTF-1^+^ cells multiplied by 100%, respectively.

### Inflammatory Cell Analysis

Five individual fields with infiltration of inflammatory cells were selected for total and differential cell counts (Supplementary Figure S3). Total cells counts were derived from counting 200 leukocytes (under 400× magnification). Differential cell counts of the inflammatory cells were expressed as the percentage number of positive staining cells/200 leukocytes × 100%. For each inflammatory cell count (e.g., CD4^+^ T cells, CD8^+^ T cells, eosinophils and neutrophils), the actual percentage number was recoded. Eosinophilic or neutrophilic infiltration was defined by eosinophils or neutrophils count being greater than 10% of the total leukocyte count, respectively (Chen et al., 2018).

### Statistical Analysis

Statistical analyses were conducted with SPSS 21.0 software (IBM, Chicago, IL, United States) and GraphPad Prism 6 (GraphPad Software, La Jolla, CA, United States). The normal distribution was tested, and the Mann-Whitney two-sided non-parametric test was used as appropriate to compare the continuous variables between two groups. Correlation analysis was performed with Spearman’s model. *P* < 0.05 was deemed statistically significant for all analyses.

## Results

### Subject Characteristics

The clinical characteristics of control subjects and bronchiectasis patients are shown in Table 1. Bronchiectasis patients yielded significantly lower percent predicted of forced expiratory volume in 1 s (FEV_1_) (mean: 81.8% vs. 105.6%, *P* < 0.01) than control subjects. 93.0% of bronchiectasis patients were never-smokers. 60.5% (*n* = 26) patients had bilateral bronchiectasis. In 17 patients with unilateral disease, 52.9% (*n* = 9) had right lung involvement. 93.0% (*n* = 40) of patients had both central and peripheral bronchiectasis, and the remainders had peripheral bronchiectasis only. The mean modified Reiff score was 6.3 among 43 bronchiectasis patients, of whom 48.8% (*n* = 21) had cystic bronchiectasis. For 43 bronchiectasis patients, the mean ± standard deviation percentage of eosinophils, neutrophils, CD4^+^ T cells and CD8^+^ T cells was 8.3 ± 5.9, 9.8 ± 5.0, 33.1 ± 9.7, and 15.8 ± 7.5, respectively. Furthermore, Eosinophilic and neutrophilic infiltrations are found in 46.5% (20/43) and 44.2% (19/43) of bronchiectasis patients, respectively.

### Aberrant TTF-1 Expression in the Dilated Bronchioles

The IHC staining shows normal peripheral airway structure in controls subjects (Figure 1C). Consistent with previous findings (Hösgör et al., 2002), negative TTF-1 staining was found in the epithelial areas of trachea (A), proximal bronchi (B) and distal bronchi (C) from healthy adult donors (Supplementary Figure S1). TTF-1 was extensively expressed in AECII and a subset of normal bronchiolar ECs (Figures 1C-a–c). By contrast, we noted significantly dilated bronchioles in bronchiectasis patients (Figure 1D). The percentage of TTF-1^+^ cells within the dilated bronchiolar epithelium was markedly lower compared with that within the control bronchioles (Figure 1D-d).

**FIGURE 2:**
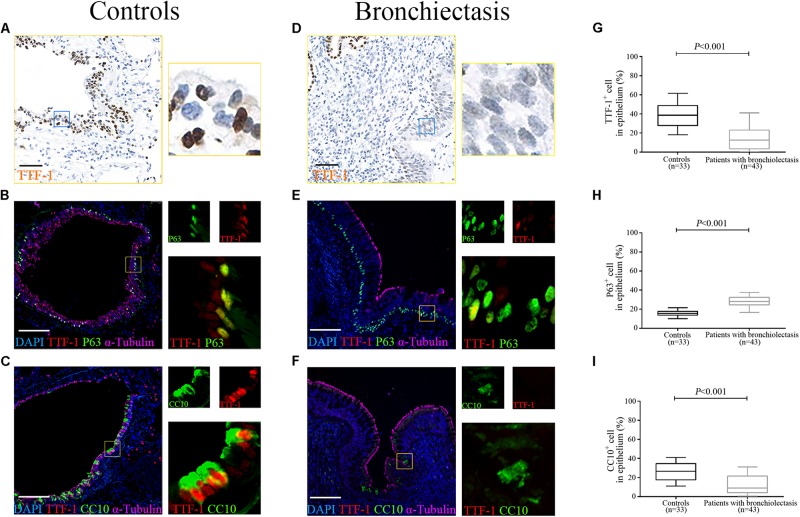
Aberrant progenitor marker expression within the dilated bronchiolar epithelium. The IF staining reveal the expression of progenitor cell markers within bronchiolar epithelium in both control subjects and bronchiectasis patients. TTF-1 (red) co-localizes with P63 (green), whereas TTF-1 (red) co-stains with CC10 (green), in a subset of ECs **(A–F)**. A significantly lower percentage of TTF-1^+^ECs and CC10^+^ECs and higher percentage of P63^+^ECs could be identified in bronchiectasis patients compared with control subjects **(G–I)**. Scale bar = 100 μm. CC10: club cell 10kDa protein; EC: epithelial cell; TTF-1: thyroid transcription factor-1.

Two patterns of aberrant TTF-1 expression dominated the sub-epithelium of dilated bronchioles: (1) columnar epithelial hyperplasia (including wall-like, canalicular, or pseudoglandular distribution) (Figure 1D-e); (2) cuboidal epithelial hyperplasia (including wall-like or canalicular distribution) (Figure 1D-f).

### Aberrant Progenitor Marker Expression Within the Dilated Bronchiolar Epithelium

The IF staining revealed the expression of progenitor cell markers within bronchiolar epithelium in both control subjects and bronchiectasis patients. TTF-1 partially co-localized with P63, whereas TTF-1 partially co-stained with CC10, in a subset of ECs (Figures 2A–F). We noted a significantly lower percentage of TTF-1^+^ECs and CC10^+^ECs and higher percentage of P63^+^ECs in bronchiectasis patients compared with control subjects (all *P* < 0.05, Figures 2G–I).

**FIGURE 3:**
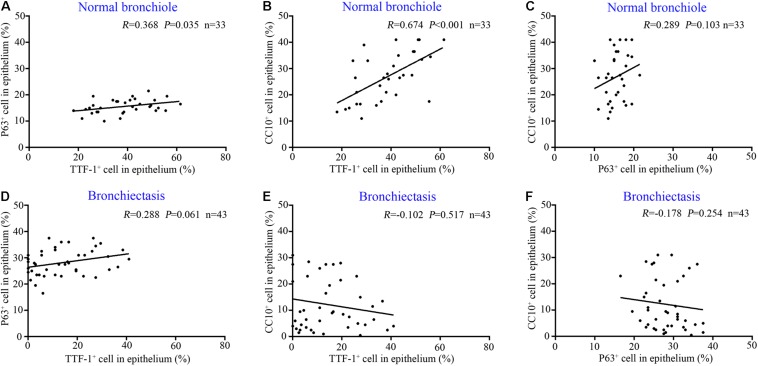
Correlation among the expression of TTF-1^+^ECs, P63^+^ECs or CC10^+^ECs in control subjects and patients with bronchiectasis. In the epithelium of normal bronchioles, there is a positive correlation between TTF-1^+^ECs and P63^+^ECs **(A)** and between TTF-1^+^ECs and CC10^+^ECs **(B)**, but not in P63^+^ECs and CC10^+^ECs **(C)**. In the epithelium of dilated bronchioles, no significant correlation is identified **(D–F)**. CC10: club cell 10 kDa protein; EC: epithelial cell; TTF-1: thyroid transcription factor-1.

In control subjects, the percentage of TTF-1^+^ECs correlated positively with that of both P63^+^ECs (Figure 3A) and CC10^+^ECs in the bronchiolar epithelium (Figure 3B), which partially co-localized with TTF-1. However, the percentage of P63^+^ECs did not correlate with that of CC10^+^ECs within the epithelium of control bronchioles (*P* > 0.05). Furthermore, there was no significant correlation between the percentage of TTF-1^+^ECs and P63^+^ECs or CC10^+^ECs in the dilated bronchiolar epithelium in bronchiectasis (both *P* > 0.05, Figures 3C–F).

### Progenitor Marker Expression Within the Bronchiole Epithelium Was Not Different When Stratified by the Key Clinical Characteristics and the Infiltration of Inflammatory Cells of Bronchiectasis

**FIGURE 4:**
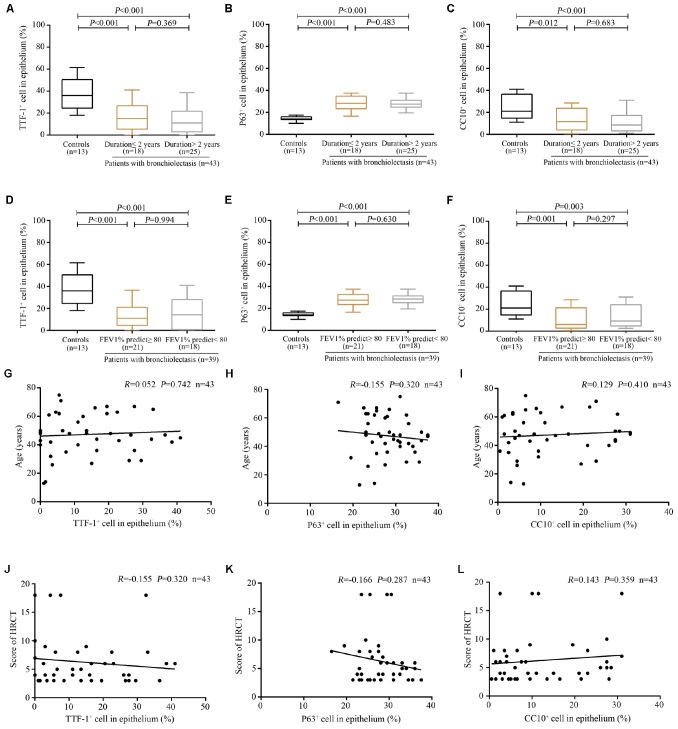
Association between the expression of TTF-1^+^ECs, P63^+^ECs or CC10^+^ECs and the clinical characteristics of patients with bronchiectasis. **(A–C)** A significantly decreased percentage of TTF-1^+^ECs and CC10^+^ECs and a higher percentage of P63^+^ECs in the dilated bronchioles are identified in bronchiectasis when stratifying patients based on the duration of symptoms (cut-off: 2 years). **(D–F)** A significantly decreased percentage of TTF-1^+^ECs and CC10^+^ECs and a higher percentage of P63^+^ECs in the dilated bronchioles are identified in bronchiectasis when stratifying patients based on the magnitude of lung function impairment (cut-off: 80% for FEV_1_ predicted %). No significant correlation between the percentage of TTF-1^+^ECs, P63^+^ECs, and CC10^+^ECs and the age **(G–I)** as well as the modified Reiff score **(J–L)**. CC10: club cell 10kDa protein; EC: epithelial cell; FEV1: first second percentage; HRCT: high-resolution computed tomography; TTF-1: thyroid transcription factor-1.

Next, we stratified bronchiectasis patients according to the key clinical metrics. None of the 13 control subjects (with benign tumors, data from 20 healthy donors were excluded because of the policy on anonymization) presented with chronic cough or abnormal lung function. Compared with control subjects, significantly decreased percentage of TTF-1^+^ECs and CC10^+^ECs and a higher percentage of P63^+^ECs were identified in the dilated bronchioles in bronchiectasis (all *P* < 0.05). However, the cell count did not differ statistically when stratified by the duration of symptoms (cut-off: 2 years) and lung function impairment (cut-off: 80% for FEV_1_ predicted %) among bronchiectasis patients (Figures 4A–F). However, the percentage of TTF-1^+^ECs, P63^+^ECs and CC10^+^ECs did not correlate with the age or the modified Reiff score (Figures 4G–L).

Overall, there was no significant association between each inflammatory cell count (e.g., CD4^+^ T cells, CD8^+^ T cells, eosinophils, and neutrophils) and the marker expression profiles of progenitor cells (TTF-1^+^ECs, P63^+^ECs, and CC10^+^ECs) of dilated bronchiole (except for the significant association between P63^+^ ECs cell count and CD8^+^ cell count) (Supplementary Figure S4). Furthermore, the percentage of epithelial progenitor cells (TTF-1, P63, and CC10) in the dilated bronchioles was not statistically different between eosinophilic and non-eosinophilic inflammation, nor between neutrophilic and non-neutrophilic inflammation (cut-off: 10% of all leukocytes) (Supplementary Figure S5).

### Aberrant Progenitor Marker Expression in the Dilated Bronchiolar Sub-Epithelium

Next, we explored the association between progenitor marker expression and cuboidal/columnar epithelial hyperplasia in the dilated bronchiolar sub-epithelium (Figures 1D-e,f). TTF-1 was stained to indicate epithelial hyperplasia in the dilated bronchiolar sub-epithelium. We also applied IF triple co-staining with α-tubulin (indicating the dilated bronchiolar epithelium), TTF-1 (indicating multiple airway progenitors) and one of the epithelial cell markers (P63 for BCs, CC10 for club cells and SPC for AECII) in the bronchiolar or alveolar epithelium of the distal airways).

**FIGURE 5:**
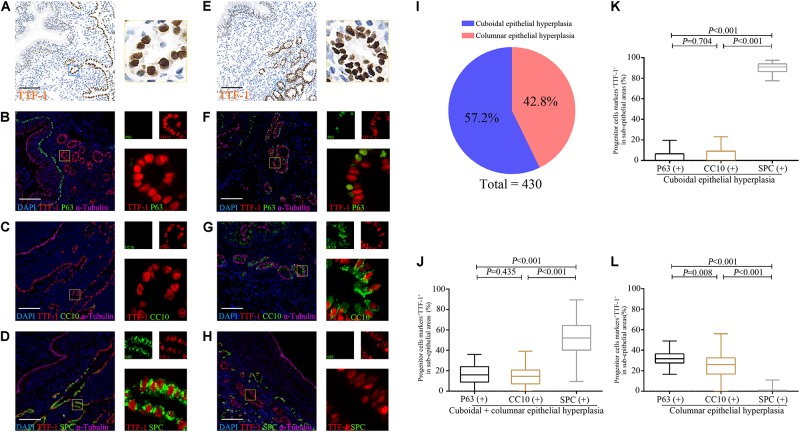
Expression of TTF-1, P63, CC10 and SPC protein in the sub-epithelium areas of the dilated bronchioles in bronchiectasis. Two major abnormal patterns are observed in the sub-epithelium areas of control and dilated bronchioles from patients with bronchiectasis with TTF-1 staining: cuboidal epithelial hyperplasia **(A–D)** and columnar epithelial hyperplasia **(E–H)**. We confirm that most progenitor markers (P63, CC10 or SPC) (green) co-localize with TTF-1 (red) **(B–D,F–H)**. We count 430 areas (10 areas per patient) with epithelial hyperplasia. 57.2% (*n* = 246) of areas present with cuboidal epithelial hyperplasia, whereas 42.8% (*n* = 184) of areas yield columnar epithelial hyperplasia **(I)**. The percentage of P63^+^TTF-1^+^, CC10^+^TTF-1^+^ and SPC^+^TTF-1^+^ are assessed in both cuboidal and columnar epithelial hyperplasia **(J)**, only for cuboidal epithelial hyperplasia **(K)** and only for columnar epithelial hyperplasia **(L)**, respectively. α-tubulin (pink) is stained as a ciliary marker for epithelium which is helpful to distinguish the sub-epithelium areas. Scale bar = 100 μm. CC10: club cell 10 kDa protein; SPC: surfactant protein C; TTF-1: thyroid transcription factor-1.

We confirmed that most epithelial markers in the distal airways (P63, CC10, and SPC) co-localized with TTF-1 in the dilated bronchiolar sub-epithelium (Figures 5A–H). We also noted remarkable inflammatory cell (i.e., lymphocytes, neutrophils) infiltration that precluded an accurate cell enumeration. In this regard, we enumerated TTF-1^+^ cells that yielded abnormal patterns of epithelial hyperplasia. We noted two main types of epithelial hyperplasia with disarrangement of TTF-1^+^ cells: cuboidal (100%) and columnar (93.0%) among the 43 bronchiectasis patients. We next counted 430 areas (10 areas per patient) with epithelial hyperplasia. 57.2% (*n* = 246) of areas presented with cuboidal epithelial hyperplasia, whereas 42.8% (*n* = 184) of areas yielded columnar epithelial hyperplasia (Figure 5I).

To evaluate sub-epithelial hyperplasia, we randomly counted 200 abnormal TTF-1^+^ cells in 5∼10 HPFs in the dilated bronchiolar sub-epithelium per patient. The median (the 1st, 3rd quartile) percentage of P63^+^TTF-1^+^, CC10^+^TTF-1^+^, and SPC^+^TTF-1^+^ cells was 16.0% (8.9, 24.0%), 14.5% (7.1, 20.8%), and 52% (40.3, 64.4%), respectively (Figure 5J).

We then evaluated cuboidal and columnar epithelial hyperplasia, respectively. For cuboidal epithelial hyperplasia with TTF-1^+^ cells, 91.0% (86.5, 94.0%) of areas co-stained with SPC, which accounted for a significantly greater percentage of cell count compared with the cells co-stained with P63 or CC10 (Figure 5K). For columnar epithelial hyperplasia with TTF-1^+^ cells, the percentage of cells co-stained with progenitor markers followed the following order: P63^+^ > CC10^+^ > SPC^+^ (Figure 5L).

**FIGURE 6:**
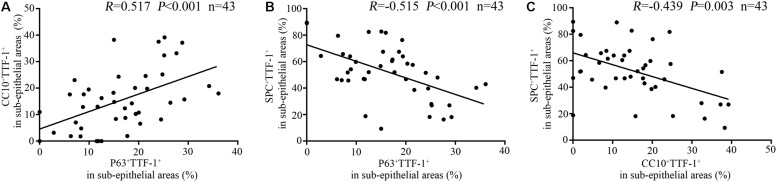
Correlation among the percentage of P63^+^TTF-1^+^, CC10^+^TTF-1^+^, and SPC^+^TTF-1^+^ cells in the sub-epithelium of the dilated bronchioles in bronchiectasis. The percentage of P63^+^TTF-1^+^ cells correlates positively with that of CC10^+^TTF-1^+^ cells **(A)**, and negatively with that of SPC^+^TTF-1^+^ cells in the dilated bronchiolar sub-epithelium **(B)**. The percentage of CC10^+^TTF-1^+^ cells also correlates negatively with that of SPC^+^TTF-1^+^ cells **(C)**. CC10: club cell 10 kDa protein; SPC: surfactant protein C; TTF-1: thyroid transcription factor-1.

**FIGURE 7:**
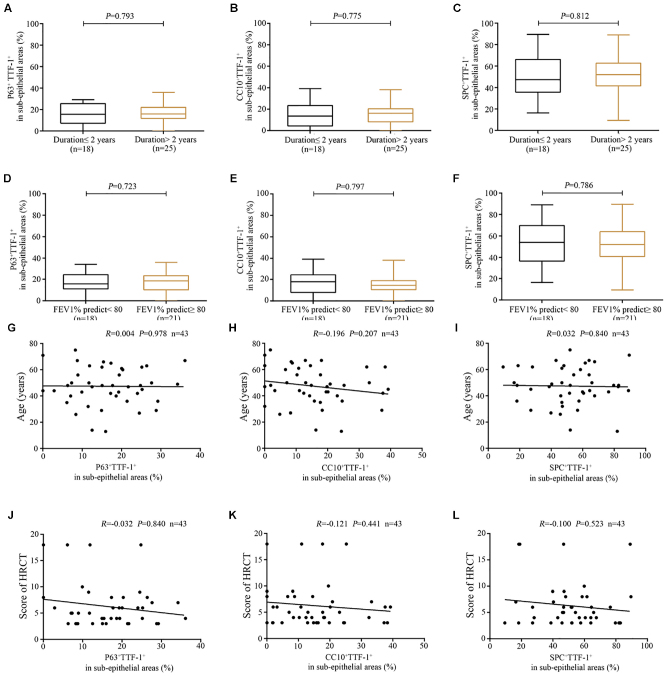
Association between the expression of P63^+^TTF-1^+^, CC10^+^TTF-1^+^, and SPC^+^TTF-1^+^ in the sub-epithelium of dilated bronchioles and the clinical characteristics of bronchiectasis. **(A–C)** A similar percentage of P63^+^TTF-1^+^, CC10^+^TTF-1^+^ and SPC^+^TTF-1^+^ in the sub-epithelium of the dilated bronchioles based on the duration of symptoms (cut-off: 2 years). **(D–F)** A similar percentage of P63^+^TTF-1^+^, CC10^+^TTF-1^+^, and SPC^+^TTF-1^+^ in the sub-epithelium of dilated bronchioles based on lung function impairment (cut-off: 80% for FEV_1_ predicted %). No significant correlation between the percentage of P63^+^TTF-1^+^, CC10^+^TTF-1^+^, and SPC^+^TTF-1^+^ and the age **(G–I)** as well as the modified Reiff score **(J–L)**. CC10: club cell 10 kDa protein; EC: epithelial cell; FEV1: first second percentage; HRCT: high-resolution computed tomography; SPC: surfactant protein C; TTF-1: thyroid transcription factor-1.

Furthermore, the percentage of P63^+^TTF-1^+^ cells correlated positively with that of CC10^+^TTF-1^+^ cells (Figure 6A), and negatively with that of SPC^+^TTF-1^+^ cells in the dilated bronchiolar sub-epithelium (Figure 6B). Moreover, the percentage of CC10^+^TTF-1^+^ cells also correlated negatively with SPC^+^TTF-1^+^ cells (Figure 6C). We also found no significant correlation between the percentage of P63^+^TTF-1^+^, CC10^+^TTF-1^+^ and SPC^+^TTF-1 + cells in the sub-epithelium and the duration of symptoms, nor was a significant correlation found with FEV_1_ % predicted, age and the modified Reiff score in bronchiectasis patients (Figure 7).

Finally, we noted there was no remarkable difference between each inflammatory cell count (e.g., CD4^+^ T cells, CD8^+^ T cells, eosinophils, and neutrophils) and the percentage of different types of sub-epithelial progenitor cells (P63^+^TTF-1^+^, CC10^+^TTF-1^+^ and SPC^+^TTF-1^+^) (Supplementary Figure S6). The percentage of different types of sub-epithelial progenitor cells was also not statistically different between eosinophilic and non-eosinophilic inflammation, nor between neutrophilic and non-neutrophilic inflammation (Supplementary Figure S7).

## Discussion

Airway ECs constitute the first barrier against pathogens and xenobiotics, and play a critical role in maintaining homeostasis (Whitsett and Alenghat, 2015). Structural and functional abnormalities of airway epithelium may dampen host defenses, immune or inflammatory responses, and repair processes, leading to progressive epithelial injury and recurrent infections in bronchiectasis (Maeda et al., 2011; Guan et al., 2018a; Nikolic, 2018).

To our knowledge, this is the first study that has systematically investigated the expression profiles of progenitor cells in peripheral airways of bronchiectasis. We have revealed abnormal cell proliferation in both epithelium and sub-epithelium of the dilated bronchioles (previously regarded as clinically silent zones) in bronchiectasis. Despite extensive validation, the vicious cycle model falls short of elucidating the pathologic changes associated with the progressive airway injury that cannot readily be reversed by antibiotic or mucolytic therapies (Polverino et al., 2017). Our findings have pointed to the critical roles of defective progenitor cell renewal, suggesting candidate targets for paradigm shift in future disease management (Graphical abstract).

In control subjects, progenitor cells in distal airway epithelium consist of BC, club cells (both in bronchioles) and AECII (in alveoli) which partially yielded TTF-1 expression. Different from the distal airway, we did not identify any TTF-1-positive cells in the normal epithelial areas of proximal airways (trachea and bronchi) from healthy donors (*n* = 4) (Supplementary Figure S1). Notably, we have identified the aberrant expression of progenitor cell markers (TTF-1, P63, and CC10) in bronchiolar epithelium in bronchiectasis. The two dominant patterns, including cuboidal epithelial hyperplasia (TTF-1 mostly co-localized with SPC, Figure 5K) and columnar epithelial hyperplasia (TTF-1 mainly co-localized with P63 or CC10, Figure 5L) in the sub-epithelium of the dilated bronchioles, indicated the dysregulated proliferation of the injured epithelium. Previous reports have documented TTF-1 expression in ECs of human fetal lungs at 11–12 weeks’ gestation. ECs of the peripheral developing airways during pseudoglandular period (weeks 12–16) and canalicular period (weeks 16–28) strongly expressed TTF-1, presenting with the morphology highly similar with the cuboidal and columnar epithelial hyperplasia as seen in our study (Kimura and Deutsch, 2009; Kaarteenaho et al., 2010). Collectively, pathologic dilatation of bronchioles may be linked to defective lung repair or regeneration.

Recent studies have documented the aberrant structure or function of ECs (e.g., ciliated cell, BC and goblet cells) in chronic airway inflammatory diseases (Guan et al., 2018b). For instance, up-regulation of P63 contributes to epithelial remodeling in nasal polyps (Zhao et al., 2017). Zuo et al. (2015) reported that p63^+^ keratin 5 (Krt5)^+^ distal airway stem cells give rise to multiple epithelial cell lineages, including bronchiolar secretory cells as well as alveolar type I and type II pneumocytes, to regenerate the distal lung in response to influenza-induced lung damage. In peripheral airways, AECII failed to repair the damaged epithelium as a result of defective proliferation, migration, and/or differentiation, which reportedly led to interstitial scarring in pulmonary fibrosis (Sisson et al., 2010). The greater type I alveolar differentiation potential in the distal airway stem cells in COPD (Wang Y. et al., 2019), coupled with the metaplastic transition to a muco-secretory phenotype in the terminal airways of patients with asthma and COPD (Jeffery, 2001), have provided further evidence of aberrant proliferation of the peripheral airways in chronic airway inflammatory diseases.

Main strengths of our study included the comprehensive profiling of progenitor cell markers that helped unravel dysregulated epithelial proliferation in the peripheral airways of bronchiectasis which have been rarely investigated. Our findings might form a novel basis for exploring airway proliferation as a therapeutic target of bronchiectasis. However, our study is limited by the inclusion of the heterogeneous etiologies of bronchiectasis, and the limited sample size from a single center. External validation of our findings is therefore warranted. The IHC staining also fell short of providing quantitative assessment of the expression levels or the activity of progenitor cell markers. We did not assess airway pathogens because of the archival tissues. Incorporation of airway infection status and inflammatory phenotypes with paired sputum or bronchoalveolar lavage samples might provide greater insights into the interaction between the host-defense and airway infection and inflammation.

In summary, we have unraveled the aberrant expression of progenitor cells in bronchiole epithelium and sub-epithelium in bronchiectasis. The dysregulated epithelial cell proliferation might represent a novel research direction and provide potential targets for future therapeutic interventions of bronchiectasis.

## Data Availability Statement

The raw data supporting the conclusions of this article will be made available by the authors, without undue reservation, to any qualified researcher.

## Ethics Statement

The studies involving human participants were reviewed and approved by the institutional review boards of The First Affiliated Hospital of Guangzhou Medical University. Written informed consent to participate in this study was provided by the participants’ legal guardian/next of kin.

## Author Contributions

NZ, D-YW, and WG: conceived the experiments. YP, YH, and XH: collection of samples. YP, AX, and SC: performed the experiments. YP, AX, and SC: data analysis. YP and WG: wrote the manuscript. NZ and D-YW: critical review and approval.

## Conflict of Interest

The authors declare that the research was conducted in the absence of any commercial or financial relationships that could be construed as a potential conflict of interest.

## Abbreviations

AECII: type II alveolar epithelial cell
BC: basal cell
CC10: club cell 10 kDa protein
DAPI: 4 ′ 6-diamidino-2-phenylindole
FEV1: first second percentage
FVC: forced vital capacity
HPF: high power field
HRCT: high-resolution computed tomography
HRP: horseradish peroxidase
IF: immunofluorescent
IHC: immunohistochemistry
SPC: surfactant protein C
TTF-1: thyroid transcription factor-1.
