# Supplementary material for: Aberrant Epithelial Cell Proliferation in Peripheral Airways in Bronchiectasis
Source: Front Cell Dev Biol. 2020 Feb 20;8:88. doi: 10.3389/fcell.2020.00088 (PMC7044270; doi:10.3389/fcell.2020.00088)
Supplement: Supplementary file 1 [file Data_Sheet_1.docx]

**LIST OF ONLINE SUPPORTING INFORMATION**

**FIGURE LEGENDS**

**Figure S1. TTF-1 staining in different histologic areas in healthy controls**

Negative TTF-1 staining in epithelial areas of trachea (A), proximal bronchi (B) and distal bronchi (C) from heathy adult donors (n=4).

Green arrow: TTF-1 positive cell (type II alveolar epithelial cell)

TTF-1: thyroid transcription factor-1.

**Figure S2.** **Overview of peripheral lung tissue in bronchiectasis**

Panoramic slice scanning (A) for peripheral lung tissue from patients with bronchiectasis, and 5 HPFs (B) for epithelial hyperplasia with disarrangement of TTF-1^+^ cells in sub-epithelium of the dilated bronchioles.

Green triangle: dilated bronchioles.

HPF: high power field; TTF-1: thyroid transcription factor-1.

**Figure S3.** **Inflammatory cell infiltration in the dilated bronchioles**

Infiltration and distribution of eosinophils (A), neutrophils (B), CD4^+^ T cells (C) and CD8^+^ T cells (D) in the dilated bronchiole.

**Figure S4. Association between the expression of TTF-1^+^ECs, P63^+^ECs or CC10^+^ECs and the infiltration of inflammatory cells in bronchiectasis**

No significant correlation between the percentage of TTF-1^+^ECs, P63^+^ECs and CC10^+^ECs and infiltration of eosinophils (A-C), neutrophils (D-F), and CD4^+^ T cells (G-I) in the dilated bronchiole. For CD8^+^ T cells, there is no significant correlation between TTF-1^+^ECs (J) and CC10^+^ECs (L), but the significant correlation with P63^+^ECs could be identified (K).

CC10: club cell 10kDa protein; EC: epithelial cell; TTF-1: thyroid transcription factor-1.

**Figure S5**. **TTF-1^+^ECs, P63^+^ECs and CC10^+^ECs of the dilated bronchioles when stratified by the number of infiltrating inflammatory cells**

A similar percentage of TTF-1^+^ECs, P63^+^ECs and CC10^+^ECs of the dilated bronchioles when stratified by the number of infiltrating eosinophils (A-C), neutrophils (D-F) (cut-off: 10% of leukocytes).

EOS: eosinophils; NEU: neutrophils.

CC10: club cell 10kDa protein; EC: epithelial cell; TTF-1: thyroid transcription factor-1.

**Figure S6. Association between the expression of P63^+^TTF-1^+^, CC10^+^TTF-1^+^ and SPC^+^TTF-1^+^ in the sub-epithelium of dilated bronchioles and the infiltration of inflammatory cells of bronchiectasis**

No significant correlation between the percentage of P63^+^TTF-1^+^, CC10^+^TTF-1^+^ and SPC^+^TTF-1^+^ in the sub-epithelium of the dilated bronchioles and the infiltration of eosinophils (A-C), neutrophils (D-F), CD4^+^ T cells (G-I) and CD8^+^ T cells (J-L).

CC10: club cell 10kDa protein; SPC: surfactant protein C; TTF-1: thyroid transcription factor-1.

**Figure S7**. **P63^+^TTF-1^+^, CC10^+^TTF-1^+^ and SPC^+^TTF-1^+^ cells in the sub-epithelium of dilated bronchioles based on inflammatory cell infiltration patterns**

A similar percentage of P63^+^TTF-1^+^, CC10^+^TTF-1^+^ and SPC^+^TTF-1^+^ in the sub-epithelium of the dilated bronchioles when stratified by the number infiltrating eosinophils (A-C) and neutrophils (D-F) (cut-off: 10% of leukocytes).

CC10: club cell 10kDa protein; EOS: eosinophils; NEU: neutrophils; SPC: surfactant protein C; TTF-1: thyroid transcription factor-1.
