# Supplementary figures and images for: Aberrant Epithelial Cell Proliferation in Peripheral Airways in Bronchiectasis
Source: Front Cell Dev Biol. 2020 Feb 20;8:88. doi: 10.3389/fcell.2020.00088 (PMC7044270; doi:10.3389/fcell.2020.00088)

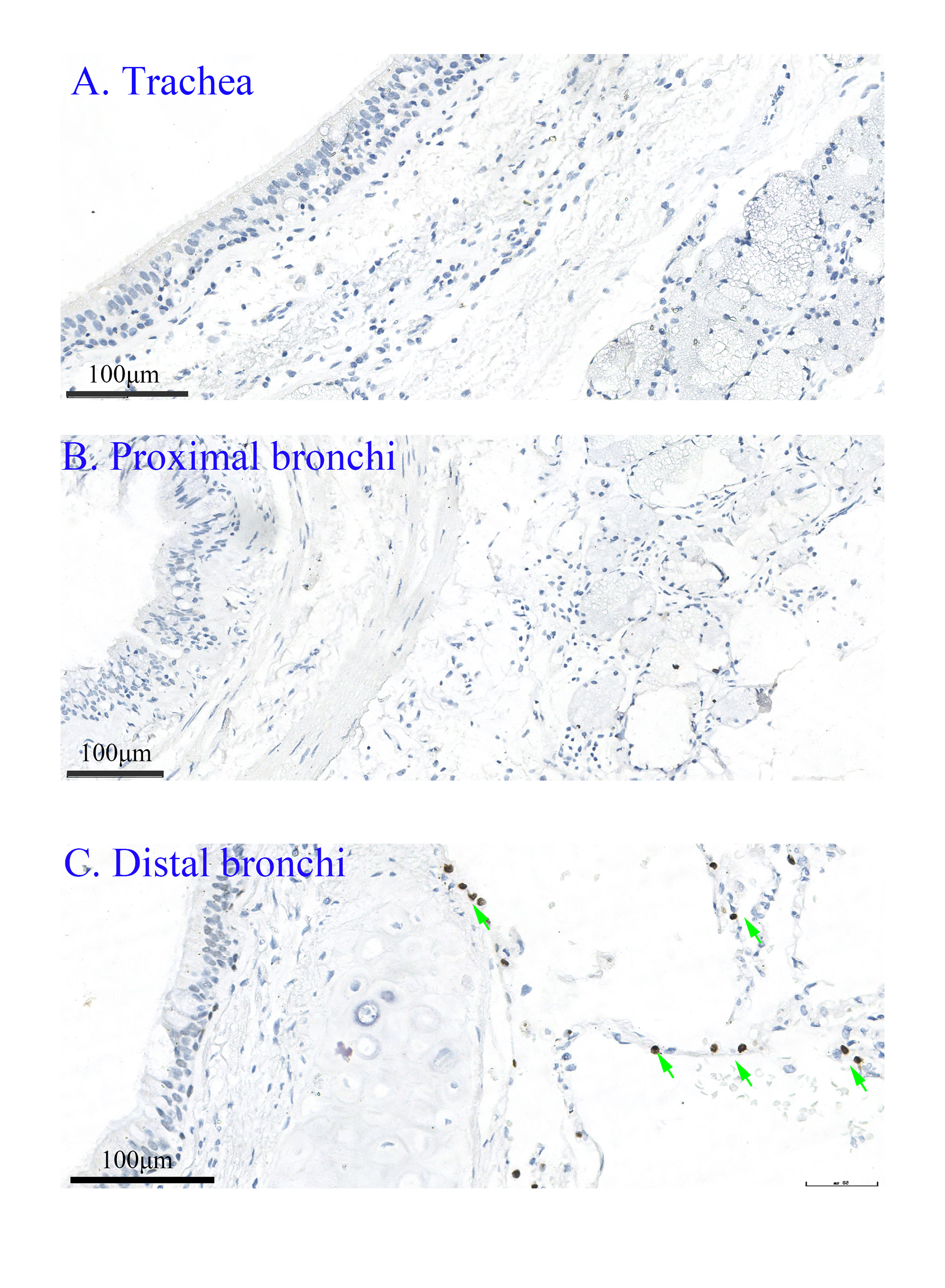

Supplement: Supplementary file 2 [file Image_1.TIF]

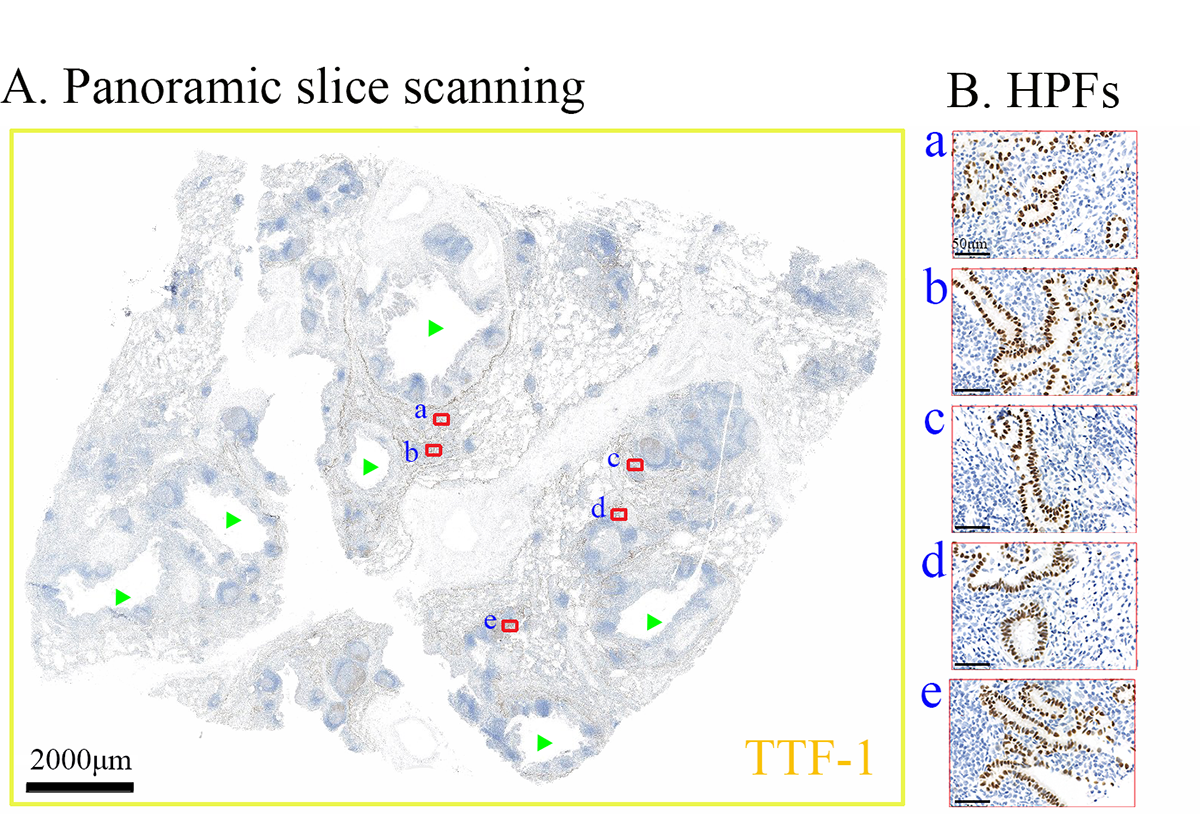

Supplement: Supplementary file 3 [file Image_2.TIF]

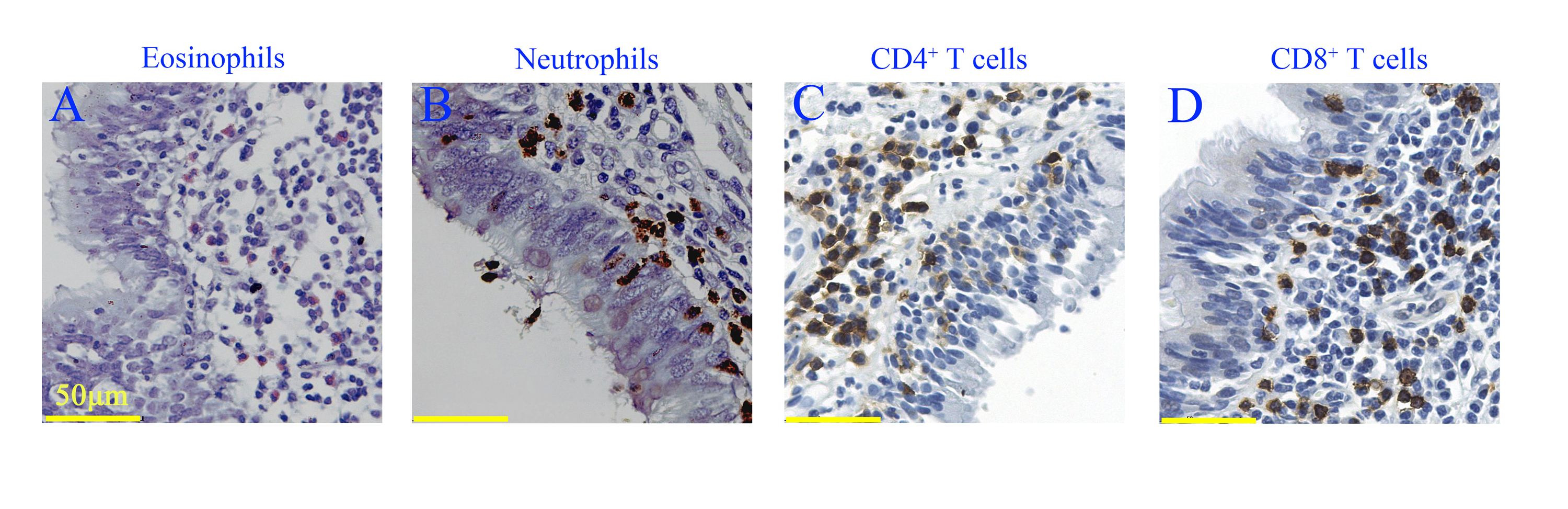

Supplement: Supplementary file 4 [file Image_3.TIF]

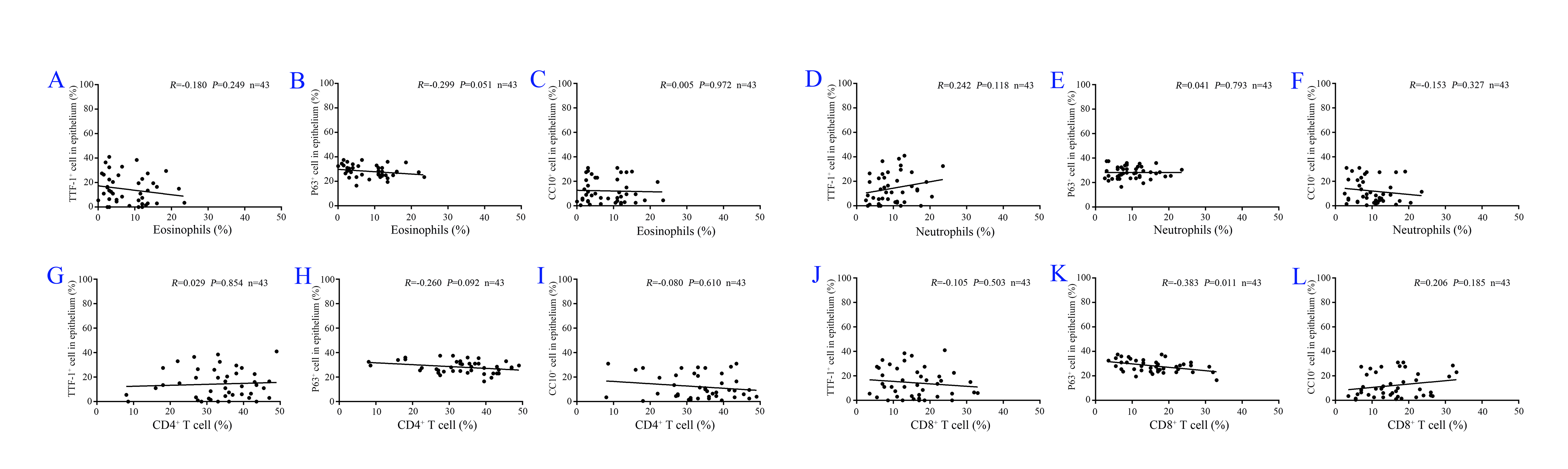

Supplement: Supplementary file 5 [file Image_4.TIF]

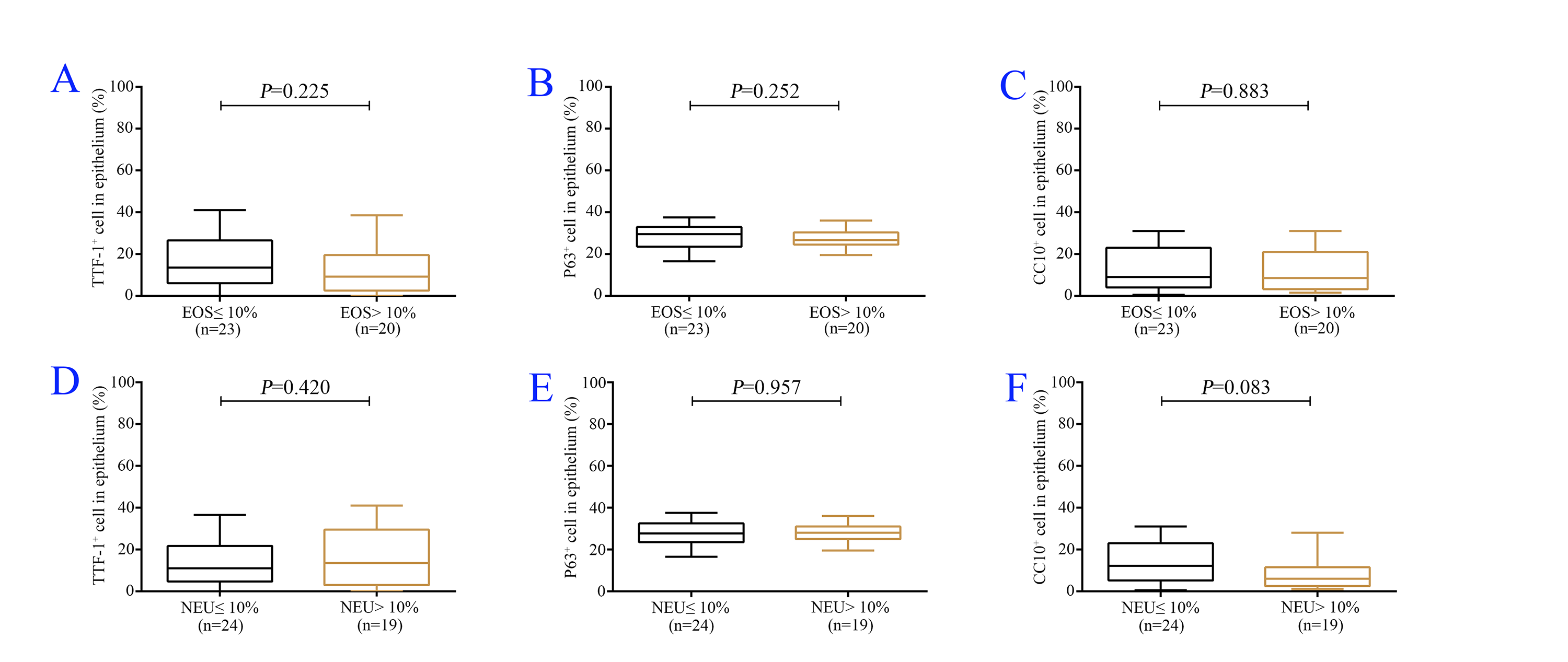

Supplement: Supplementary file 6 [file Image_5.TIF]

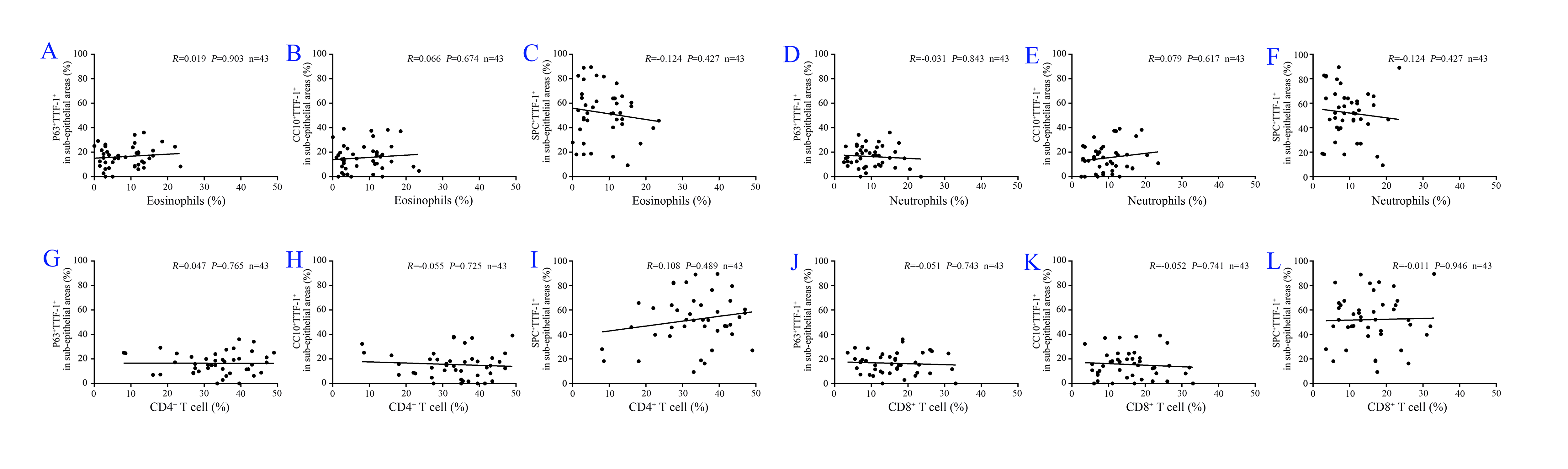

Supplement: Supplementary file 7 [file Image_6.TIF]

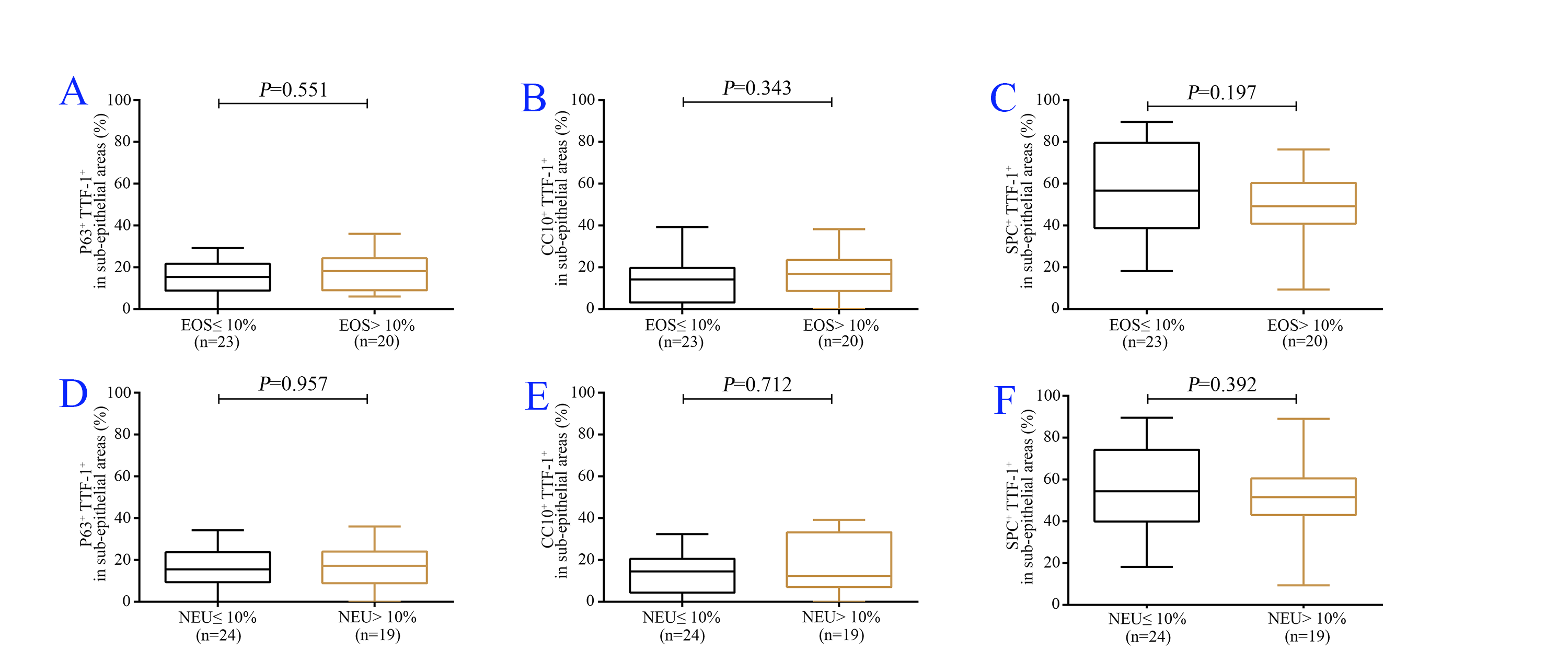

Supplement: Supplementary file 8 [file Image_7.TIF]
